# Economic burden of musculoskeletal disorders in Tanzania: results from a community-based survey

**DOI:** 10.1136/bmjopen-2024-087425

**Published:** 2025-01-15

**Authors:** Manuela Deidda, Eleanor Grieve, Stefanie Krauth, Ping-Hsuan Hsieh, Nateiya Yongolo, Stefan Siebert, Jo Halliday, Sanjura Mandela Biswaro, Kajiru Kilonzo, Richard Walker, Clive Kelly, Elizabeth F Msoka, Kiula Kiula, Blandina Mmbaga, Emma McIntosh

**Affiliations:** 1 Health Economics and Health Technology assessment, School of Health and Wellbeing, University of Glasgow, Glasgow, UK; 2 General Practice and Primary Care, School of Health and Wellbeing, University of Glasgow, Glasgow, UK; 3 School of Allied and Public Health Professions, Canterbury Christ Church University, Canterbury, UK; 4 School of Pharmacy, National Defense Medical Center, Taipei, Taiwan; 5 Kilimanjaro Clinical Research Institute, Moshi, Tanzania; 6 School of Infection & Immunity, University of Glasgow, Glasgow, UK; 7 School of Biodiversity, One Health and Veterinary Medicine, College of Medical Veterinary and Life Sciences, University of Glasgow, Glasgow, UK; 8 Kilimanjaro Christian Medical University College, Moshi, Tanzania; 9 Newcastle University, Newcastle upon Tyne, UK

**Keywords:** HEALTH ECONOMICS, RHEUMATOLOGY, Surveys and Questionnaires

## Abstract

**Abstract:**

**Objectives:**

To identify, measure and value the economic burden of musculoskeletal (MSK) disorders in the Kilimanjaro region, Tanzania.

**Design:**

Community-based cross-sectional survey (undertaken between January and September 2021).

**Setting:**

Hai district, Kilimanjaro, Tanzania.

**Participants:**

Households resident in the Hai district.

**Methods:**

A two-stage cluster sampling was used to select a representative sample of all Hai district residents. Clinical screening tools were used to identify and measure MSK disorders through a tiered approach. An economic questionnaire measuring healthcare costs, out-of-pocket costs, absenteeism, presenteeism and work productivity loss was administered to those with likely MSK disorders and selected controls (individuals without MSK disorders, matched by age and gender). Resource use was valued using country-specific costs. Two-part model regressions were fitted. A descriptive analysis of catastrophic expenditure was also conducted.

**Main outcome measure:**

Healthcare costs, productivity costs and total costs.

**Results:**

Annual average productivity and healthcare costs were, respectively, 3.5 and 3 times higher for those with likely MSK disorders than controls. Productivity costs of individuals with MSK disorders were Int$487 vs Int$132 in the control group (difference: Int$355, 95% CI Int$222 to Int$488). Healthcare costs in those with MSK were Int$269 vs Int$88 in the control group (difference: Int$181, 95% CI Int$34 to Int$327). The difference in terms of out-of-pocket expenses was economically substantial in magnitude, although not statistically significant.

**Conclusion:**

The evidence will be used to inform policies addressing MSK disorders, by promoting the design of interventions, service provision, health promotion and awareness activities at local, regional and national level.

**STRENGTHS AND LIMITATIONS OF THIS STUDY**

- A two-stage sampling strategy was used to identify a representative sample of residents in the Hai district.
- An economic questionnaire, tailored to the Tanzania context, captured healthcare costs, out-of-pocket costs, absenteeism, presenteeism and work productivity loss and was administered to those with likely musculoskeletal (MSK) disorders and selected controls.
- A robust econometric approach (two-part expenditure model) was used to predict costs for those with MSK disorders as compared with controls.
- The high percentage of missing responses in key variables did not allow a full estimation of catastrophic expenditures.

## Introduction

 Musculoskeletal (MSK) disorders affect joints, bones and/or other supporting tissues of the locomotor system and are associated with pain, limitations in mobility and impediment in performing daily activities.^1 2^ The global burden of disease survey has identified MSK conditions as one of the major causes of non-traumatic disability and the fourth most common cause of disability.^3^ These conditions pose a huge burden on the individual, healthcare service and society in general, in terms of direct and indirect costs and impaired quality of life for patients and their caregivers,^4^ thus representing a major societal issue. This is especially true for low-resource settings, which appear to have a similar prevalence of MSK disorders to high-resource settings, but experience a higher burden of these diseases due to limited access to care, poverty and sociocultural beliefs which might hinder a timely diagnosis,^5^ and potential loss of sources of income which could be severe considering the high prevalence of manual labour and absence of a welfare safety net. Furthermore, the rapid population growth, ageing and increase of non-communicable disease (NCD) risk factors (such as smoking, drinking, poor diet and sedentary behaviour) are contributing to an increase in the prevalence and burden represented by MSK conditions, thus making these conditions a threat to population health.^6^ Considering this, a campaign led by the United Nations and the WHO aims to increase the awareness of MSK disorders, prompting prevention and education initiatives.^6^ However, so far, most global NCD initiatives still do not include MSK conditions.^7^

In sub-Saharan Africa, there is almost a complete lack of evidence on the economic impact of MSK conditions among the general adult population. In Tanzania, a lack of data exists on the prevalence, quality of life, economic and societal impact of MSK disorders. So far, a lack of knowledge on the full impact of MSK conditions has resulted in these conditions being neglected. However, considering the potential burden represented by these diseases, measuring their full impact in terms of clinical, economic, societal and quality of life impacts on people’s day-to-day lives is needed to inform policy responses and healthcare planning, shifting limited resources to the prevention and management of MSK conditions, thus limiting the burden of MSK-caused disability. This is especially important in Tanzania, as although the country is progressing towards universal health coverage (UHC), it still faces significant financial and human resource constraints^8 9^ and strongly relies on out-of-pocket health expenditure.^10^

This paper aims at robustly estimating the economic burden of MSK disorders in the community setting, in terms of direct medical costs (eg, inpatient and outpatient visits, visits to faith healers and out-of-pocket expenses), indirect costs (absenteeism and presenteeism) and catastrophic expenditures.

## Methods

### Community survey

A community-based cross-sectional survey was undertaken between January 2021 and September 2021 in the Kilimanjaro region of Tanzania. We selected households using a two-stage random cluster sampling strategy.^11^ In the first stage, 12 villages were selected with replacement and probability proportional to the size. In the second stage, 70 households per village were randomly selected.

Overall, 1105 households were approached; 45 households declined participation and 40 households were not reached. Within 1020 participating households, 2982 individuals were eligible for participation. After excluding those who could not be reached and those who declined participation, a sample of 2632 individuals went through a tiered process.

Clinical screening tools, including the Gait Arms Legs Spine (GALS)^12^ and Regional Examination of the Musculoskeletal System (REMS),^10^ were used to screen people with likely MSK diseases and possible arthritis. In the first tier, participants underwent GALS examination. Participants who were positive on GALS screening (222) underwent subsequent REMS (tier 2). 154 participants were positive on REMS assessment (REMS+, implying confirmed MSK disorders). Controls were selected from the GALS− group and matched by age and gender in a 1: 3 ratio. REMS+ and controls were administered a detailed health economics questionnaire. Overall, the study included 153 individuals with likely MSK disorders (REMS+) and 441 controls.

Overall, the study included 153 individuals with likely MSK disorders (REMS+) and 441 controls.

Full details of the study are reported elsewhere.^11 13^

### Patient and public involvement

Key stakeholders at community, district and policy levels were involved in the reporting and dissemination plans of this research.^14^ Village leaders and enumerators were involved in the design and conduct of the community survey. A communication plan with all stakeholders was developed, involving village leaders and enumerators involved with the community survey, medical officers and healthcare providers and policy-makers.

### The health economics questionnaire

A bespoke questionnaire was developed following standard guidance^15^ to collect households costs, in collaboration with colleagues from Tanzania, using and adapting existing instruments^16^,^19^ to address our research question. The questionnaire included five sections (labour, health, income, wealth and other) with the aim of characterising our sample in terms of demographic, clinical and financial characteristics. Considering a broad, societal perspective, we captured the broad health and societal impact of MSK, such as paid and unpaid work productivity loss, inpatient stays and outpatient visits, hospital travel costs, household income (ie, main source of cash income and income range); household wealth (household inventory listing the assets and land owned by the household), financial hardships (eg, difficulty to make ends’ meet and loans). All eligible individuals within selected households in the MSK and control group responded to questions on labour, productivity, health and healthcare utilisation. Questions on income and wealth were answered by the person identified as the financial respondent (ie, the person who has the final decision regarding the household finances).

### Resource use and unit costs

The cost for each individual participant in the study was calculated by multiplying their reported use of healthcare resources by the associated unit cost. Absenteeism was measured as the number of days of work lost because of health reasons. The impact of health problems on paid work (presenteeism) and unpaid work (ie, home-based daily activities) was measured using a 10-point Visual Analogue Scale.^19^ Total loss of productivity costs (including absenteeism, presenteeism and unpaid work) was valued using the Tanzanian average (monthly) wage, following the opportunity cost approach.^20^ The 2017 Tanzanian National Health Insurance Fund (NHIF) unit costs were used to value outpatient and hospital stays.^21^ Travel costs to the hospital were self-reported by participants. The opportunity cost of travel was also estimated using the average wage. Additionally, some cost categories were self-reported by the individuals, including out-of-pocket expenses, (ie, the amount of money paid for medical expenses directly by the patient, such as prescription medicines, tests, consultation and inpatient fees), total cost of visits to traditional healer or faith healer, total cost of hospitalisation or admission to medical facilities. All costs were inflated to 2020 values, scaled to 1 year and converted to international dollars using the purchasing power parity exchange rate.^22^ Online supplemental table 1 summarises the resource use data collected in the study, the unit cost estimates employed and their sources.

### Econometric model

#### Healthcare and productivity costs

A preliminary descriptive analysis compared individuals with MSK (REMS+) and matched controls, in terms of demographic and clinical characteristics, using t-tests. A two-part expenditure model was used to predict productivity, healthcare and out-of-pocket expenditure (OOE) for individuals with MSK disorders versus controls. The two-part model^23^ explicitly considers the large proportion of zero-cost observations for individuals who have not used any healthcare resource or did not experience any absence from work in the given time period.

The first part of the model consisted of a logistic regression model to estimate the probability of having any type of expenditure. The second part of the model used a generalised linear model (GLM) with a gamma distribution and a log link to estimate the total costs conditional on individuals with positive expenditures. Using a GLM allows modelling of potential skewed distribution of costs. The modified Park test was conducted to choose the best family, while a battery of test (Pearson correlation tests, Pregibon link test and modified Hosmer and Lemeshow test) were used to guide the choice of the best link function. SEs were adjusted for clustering at household and village level. All analyses were performed using Stata V.17.

As the analysis considered both direct medical costs (ie, inpatient and outpatient stays) as well as indirect and societal costs (ie, patients’ transport costs, impact on income and labour), the two-part model was run with four different dependent variables:

1. Loss of productivity costs (P_Cost) is defined as the sum of the costs of absenteeism and presenteeism (effect on work-related activities, plus the effect on daily activity not including work-related activities).
2. Healthcare costs (HC_Cost1) is defined as the sum of total costs for outpatient visits, visits to faith/traditional healer, hospital overnight stays (calculated multiplying the number of admissions by the NHIF unit cost), self-reported actual transport cost.
3. OOE (including prescription medicines, tests, consultation and inpatient fees).
  A sensitivity analysis considered two alternative definitions of healthcare costs, specifically:
  - HC_Cost2: sum of total outpatient visits, visits to faith/traditional healer, hospital overnight stays (self-reported, instead of calculated as in HC_Cost1), self-reported actual transport cost.
  - HC_Cost3 sum of total outpatient, visits to faith/traditional healer, hospital overnight stays (calculated multiplying the number of admissions by the NHIF unit cost), opportunity cost of travel (calculated as travel time, multiplied by the minimum wage, rather than self-reported as in HC_Cost2).

The primary explanatory variable categorised individuals into MSK condition (REMS+) or control groups. Regressions were also adjusted by demographics (age, age squared and gender), socioeconomic (religion, education, occupation and marital status) and clinical/lifestyle (family members who had experienced joint pain, smoking and drinking habits, pregnancies, diagnosis of diabetes) variables.

Online supplemental table 2 lists the survey questions which were used to generate the dependent and explanatory variables. Analyses were performed to assess the extent of missingness, as well as the missing data mechanism. The 5% threshold rule of thumb suggests that complete-case analysis results are reliable only if the percentage of missing data is below the 5% threshold.^24^ Multiple imputation procedures using chained equations were used to impute missing data separately for each group (MSK/controls), creating 40 imputed datasets.^25^ The main analysis was performed on the multiply imputed dataset, whereas the complete case analysis (including survey participants with completed data on the explanatory and dependent variables) is presented as sensitivity analysis.

We analysed catastrophic expenditures, using the ‘ability to pay’ definition (ie, health spending exceeding 10% of total household resources^26^).

Full details on the methods used to estimate catastrophic expenditures have been presented in online supplemental appendix 6.

## Results

### Healthcare and productivity costs

Overall, there were a total of 594 respondents (153 MSK; 441 controls). In 99 households, 2 individuals were selected from the same household; in 5 households, 3 individuals were selected from the same households. In the households where more than one individual was selected, all household members were found as having MSK disorders in 60 instances; in 10 households, all household members did not have MSK. Online supplemental table 3 shows the percentage of missing data, by group (MSK/control), for participant characteristics and total costs (considering the specifications of costs defined above). The large proportion of data lost for the complete-case analysis strengthen the rationale for using the multiple imputation data sets in the base case. The results using the complete case (345 participants in the complete case analysis: 60 MSK+; 285 controls) are presented for comparison.

Online supplemental table 4 shows the number of those who reported zero costs. This ranged from 35% to 69%, thus supporting the use of the two-part model.

Table 1 shows descriptive statistics (mean costs) of participants, by cost category and MSK/control group (40-imputed datasets). REMS+participants had higher costs than controls. The difference was significant for productivity costs, out-of-pocket expenses and healthcare costs (HC_Cost3). While the summary statistics (table 1) are reported for completeness, skewness of costs and the large percentage of zeros required the use of mean values predicted using two-part model regressions, controlling for participants’ characteristics.

**Table 1:** Mean costs, by MSK/control group, multiple imputed dataset

**Mean costs, by MSK/control group (Int$, 2020)**				
	MSK (n=153)	Controls (n=441)	Total (n=594)	Difference
Loss of productivity costs(absenteeism+presenteeism)	594.9	121.6	243.5	473.3***
Healthcare costs (1) total cost for outpatient visits+visits to faith/traditional healer+hospital overnight stays+self-reported actual transport cost	205.9	99.2	126.7	106.7
Out-of-pocket expenditures	189.5	90.2	115.7	99.3**
Healthcare costs (2) total cost for outpatient visits+visits to faith/traditional healer+hospital overnight stays (self-reported)+self-reported actual transport cost	199.5	91.7	119.5	107.8
Healthcare costs (3) total cost for outpatient visits+visits to faith/traditional healer+hospital overnight stays+opportunity cost of travel	2067.2	494.7	899.7	1572.5*

Significance level: *p<0.1; **p<0.05; ***p<0.01.

All costs were inflated to 2020 values, scaled to 1 year and converted to international dollars using the purchasing power parity exchange rate.

MSKmusculoskeletal

Regression results for both two-part modelling parts are presented in table 2. The variable MSK is a dummy taking value 1 if the participant has MSK conditions, (ie, REMS+), 0 if they are in the control group. As shown in table 2, the estimated coefficients in the logit equation for MSK are positive and significant at 1% for all the dependent variables, indicating that participants with likely MSK disorders have a significantly higher probability of incurring loss of productivity costs, healthcare costs and out-of-pocket expenses than controls. The GLM part of the equation shows that, conditional on spending any amount, REMS+participants are more likely to sustain higher productivity loss and healthcare expenditure costs. However, the coefficient associated with MSK is not significant for OOE, showing that, conditional on having positive out-of-pocket expenses, there is no statistically significant difference in the magnitude of costs between MSK group and control groups.

**Table 2:** Regression results: probability of resource utilisation and cost estimation, multiply imputed database

Dependent variable	Loss of productivity costs		Healthcare costs (1)		Out-of-pocket expenses	
Covariates	First part (Logit)	Second part (GLM)	First part (Logit)	Second part (GLM)	First part (Logit)	Second part (GLM)
	Coefficients (SE)		Coefficients (SE)		Coefficients (SE)	
MSK disorders	4.061***	0.816***	0.759***	0.631**	1.696***	−0.267
	(−1.011)	(−0.157)	(−0.243)	(−0.278)	(−0.269)	(−0.303)
Age	0.070	0.037	0.045	0.084	0.056	−0.136
	(−0.061)	(−0.034)	(−0.044)	(−0.052)	(−0.055)	(−0.089)
Age squared	−0.0003	−0.0003	−0.0003	−0.0008**	−0.0004	0.0008
	(−0.0005)	(−0.0003)	(−0.0003)	(−0.0004)	(−0.0004)	(−0.0006)
Gender: male	−0.575	−0.561	−0.513	−1.286**	−0.564	−0.519
	(−0.665)	(−0.342)	(−0.772)	(−0.512)	(−0.691)	(−1.006)
Religion: Muslim	0.311	−0.136	0.121	−0.544*	0.149	0.173
	(−0.330)	(−0.174)	(−0.263)	(−0.321)	(−0.325)	(−0.394)
Education: middle	−0.0146	−0.0774	0.305	0.373	0.0738	−0.347
	(−0.405)	(−0.228)	(−0.384)	(−0.323)	(−0.416)	(−0.525)
Education: higher	0.428	−0.0988	0.977*	−0.133	−0.635	0.877
	(−0.584)	(−0.261)	(−0.565)	(−0.500)	(−0.778)	(−0.944)
Job status: manual occupation	−0.427	0.326*	0.0896	0.376	0.0656	0.568
	(−0.373)	(−0.170)	(−0.365)	(−0.403)	(−0.389)	(−0.510)
Job status: not working	−1.178	0.936**	0.862*	0.948	1.151*	0.843
	(−1.344)	(−0.477)	(−0.517)	(−0.583)	(−0.619)	(−0.665)
Civil status: married/cohabiting	−0.466*	−0.229	−0.116	0.393	0.0958	−0.637*
	(−0.279)	(−0.165)	(−0.237)	(−0.253)	(−0.247)	(−0.361)
Family members experience of joint pain	0.311	0.374	0.683**	−0.125	0.495	0.511
	(−0.665)	(−0.268)	(−0.345)	(−0.461)	(−0.455)	(−0.419)
Smoking habits: former smoker	0.0453	0.186	0.371	0.657	−0.0766	0.146
	(−0.455)	(−0.242)	(−0.406)	(−0.403)	(−0.415)	(−0.510)
Smoking habits: current smoker	−0.38	0.309	0.387	−0.153	0.128	0.326
	(−0.463)	(−0.279)	(−0.525)	(−0.351)	(−0.470)	(−0.432)
Drinking habits: former drinker	0.00294	−0.177	−0.0157	−0.336	−0.794**	0.273
	(−0.332)	(−0.194)	(−0.293)	(−0.298)	(−0.321)	(−0.382)
Drinking habits: current drinker	−0.154	−0.531***	−0.539*	−1.004***	−0.335	0.183
	(−0.280)	(−0.164)	(−0.282)	(−0.316)	(−0.278)	(−0.333)
Pregnancies	0.408	0.575*	0.855	1.842***	0.731	0.094
	(−0.617)	(−0.299)	(−0.734)	(−0.497)	(−0.643)	(−0.933)
Diagnosis of diabetes	0.145	−0.405*	1.307***	0.486	1.298**	0.504
	(−0.524)	(−0.208)	(−0.446)	(−0.303)	(−0.507)	(−0.490)
Constant	−1.724	4.202***	−3.471**	3.390*	−3.208*	10.46***
	(−1.904)	(−1.137)	(−1.430)	(−1.778)	(−1.721)	(−3.050)
Observations	594	594	594	594	594	594

Significance level: *p<0.1; **p<0.05; ***p<0.01.

Productivity costs include the cost of absenteeism and the cost of presenteeism. Healthcare cost (1) is defined as the sum of total cost for outpatient visits, visits to faith/traditional healer, hospital overnight stays (calculated multiplying the number of admission by the NHIF unit cost), self-reported actual transport cost; out-of-pocket expenditure include prescription medicines, tests, consultation and inpatient fees.

GLMgeneralised linear modelMSKmusculoskeletalNHIFNational Health Insurance Fund

Looking at the other covariates associated with productivity costs, the logit part of the model shows that married/cohabiting individuals have a lower probability of incurring loss of productivity costs. Conditional on incurring some costs, manual workers and those not working sustain higher costs than non-manual workers. Being a current drinker, as well as having a diagnosis of diabetes, is associated with higher productivity costs.

Having a diabetes diagnosis or family members who have experienced joint pain are positively associated with the likelihood of having positive (non-zero) healthcare costs. Conditional on having positive costs, males incur lower costs than females. Also, women who had previous pregnancies have higher healthcare costs than those who have not. Age is positively (but non-linearly) associated with the amount of healthcare expenditure. Those who report being current drinkers report lower healthcare (HC_Cost1) and out-of-pocket costs than abstainers. Diabetes significantly and positively impacts the probability of incurring OOE.

Table 3 shows the estimated yearly mean annual cost (considering all the cost categories) per individual. Individuals with MSK disorders have on average, 3.5 times higher productivity costs and 3 times higher healthcare costs than matched controls. Out-of-pocket expenses of participants who are in the MSK group are 1.8 times higher than controls, showing an economically important (ie, large in magnitude), although not statistically significant, difference.

**Table 3:** Average annual cost, per participant, by MSK/control

Predicted costs (multiply imputed dataset analysis)		
Average annual cost/individual, by MSK status		
Group	Productivity costs (Int$, 2020)	95% CIs
Control	132	(97 to 166)
REMS+	487	(352 to 621)
Difference	355	(222 to 488)
	**Healthcare costs (1) (Int$, 2020)**	
Control	88	(43 to 132)
REMS+	269	(114 to 423)
Difference	181	(34 to 327)
	**Out-of-pocket expenses (Int$, 2020)**	
Control	102	(45 to 159)
REMS+	189	(56 to 322)
Difference	87	(−32 to 207)

Notes: All costs were inflated to 2020 values, scaled to 1 year and converted to international dollars using the purchasing power parity exchange rate.

MSKmusculoskeletalREMSRegional Examination of the Musculoskeletal System

Table 4 shows the regression results for the alternative healthcare cost definitions (variables HC_cost2 and HC_cost3), while table 5 shows the estimated marginal effects. Using both dependent variables, participants with MSK are more likely than controls to incur positive costs. A positive and significant impact of confirmed MSK disorders on the magnitude of costs is observed when self-reported hospital costs are used (definition HC_cost2), whereas replacing actual travel cost with the opportunity cost of travel the effect is negative (but not significant). Online supplemental table 5 shows similar results in terms of the estimated yearly mean annual cost per individual, for the complete-case scenario.

**Table 4:** Regression results: probability of resource utilisation and cost estimation, multiply imputed database

Dependent variables	Healthcare costs (2)		Healthcare costs (3)	
Covariates	First part (logit)	Second part (GLM)	First part (logit)	Second part (GLM)
	Coefficients (SE)		Coefficients (SE)	
MSK disorders	0.707***	0.707***	0.762***	−0.416
	(−0.243)	(−0.257)	(−0.243)	(−0.257)
Age	0.0506	0.0614	0.0451	−0.0779
	(−0.044)	(−0.058)	(−0.044)	(−0.083)
Age squared	−0.0003	−0.0007	−0.0003	0.0008
	(−0.0003)	(−0.0004)	(−0.0003)	(−0.0006)
Gender: male	−0.499	−1.156**	−0.506	−1.814***
	(−0.778)	(−0.517)	(−0.773)	(−0.628)
Religion: Muslim	0.189	−0.724**	0.111	0.890**
	(−0.257)	(−0.338)	(−0.263)	(−0.416)
Education: middle	0.269	0.407	0.307	−0.214
	(−0.381)	(−0.337)	(−0.384)	(−0.378)
Education: higher	0.938*	−0.404	0.979*	−0.948**
	(−0.563)	(−0.551)	(−0.564)	(−0.403)
Job status: manual occupation	0.145	0.212	0.0874	0.859***
	(−0.359)	(−0.398)	(−0.366)	(−0.318)
Job status: not working	0.832	1.022*	0.861*	0.0305
	(−0.511)	(−0.603)	(−0.517)	(−0.483)
Civil status: married/cohabiting	−0.0513	0.537**	−0.117	0.299
	(−0.234)	(−0.237)	(−0.237)	(−0.251)
Family members experience of joint pain	0.640*	−0.0141	0.679**	−0.411
	(−0.344)	(−0.458)	(−0.346)	(−0.393)
Smoking habits: former smoker	0.329	0.579	0.37	−0.667
	(−0.403)	(−0.436)	(−0.407)	(−0.461)
Smoking habits: current smoker	0.339	−0.300	0.384	−0.279
	(−0.511)	(−0.422)	(−0.521)	(−0.623)
Drinking habits: former drinker	−0.0332	−0.338	−0.0172	0.161
	(−0.288)	(−0.312)	(−0.293)	(−0.376)
Drinking habits: current drinker	−0.555**	−1.010***	−0.542*	−0.247
	(−0.279)	(−0.356)	(−0.283)	(−0.349)
Pregnancies	0.938	1.682***	0.855	1.729***
	(−0.741)	(−0.529)	(−0.734)	(−0.569)
Diagnosis of diabetes	1.223***	0.691**	1.302***	−0.396
	(−0.445)	(−0.312)	(−0.445)	(−0.373)
Constant	−3.743**	4.031**	−3.467**	8.714***
	(−1.459)	(−2.024)	(−1.430)	(−2.686)
Observations	594	594	594	594

Significance level: *p<0.1; **p<0.05; ***p<0.01.

Healthcare cost is defined as the sum of total outpatient visits, visits to faith/traditional healer, hospital overnight stays (self-reported), self-reported actual transport cost. Healthcare cost (3) is defined as the sum of total outpatient, visits to faith/traditional healer, hospital overnight stays, opportunity cost of travel).

GLMgeneralised linear modelMSKmusculoskeletal

**Table 5:** Average annual cost, per participant, by REMS+/control

Predicted costs (multiply imputed dataset analysis)		
Average annual cost/individual, by MSK status		
Group	Healthcare costs (2) (Int$, 2020)	95% CIs
Control	83	(12 to 288)
REMS+	262	(111 to 412)
Difference	179	(42 to 315)
	**Healthcare costs (3) (Int$, 2020)**	
Control	725	(340 to 1109)
REMS+	813	(360 to 1264)
Difference	88	(−377 to 553)

Notes: All costs were inflated to 2020 values, scaled to 1 year and converted to international dollars using the purchasing power parity exchange rate.

MSKmusculoskeletalREMSRegional Examination of the Musculoskeletal System

Results on catastrophic expenditures (online supplemental appendix 6) show that around a quarter of those with MSK disorders incurred catastrophic expenditure versus approximately a1/10 of the controls.

## Discussion

This study identified, measured and valued the economic burden that MSK disorders impose on people living with these conditions in Tanzania. To our knowledge, this is the first time that this has been described in a community setting in sub-Saharan Africa. Using a robust two-stage sampling strategy, we selected a representative sample of the population, and, following a tiered approach to clinical classification of MSK disorders, we characterised the sample using GALS and REMS clinical tools to identify suspected and confirmed MSK abnormalities, respectively. The econometric approach has allowed a robust estimation of all the cost categories, while allowing for explicit modelling of the zero costs and considering the functional form, controlling for relevant demographic, clinical and lifestyle covariates.

Our findings highlight that those with MSK disorders experience significant detrimental economic impacts: REMS+individuals have approximately 2–3 times higher healthcare costs compared with those who do not have an MSK disorder. Indirect costs (absenteeism and presenteeism) are significantly higher for participants with MSK than controls. Furthermore, in consideration of the significant proportion of healthcare expenditure as a share of current health expenditure,^10^ we specifically included a question to investigate out-of-pocket expenditures. Although we did not find a statistically significant difference between participants with MSK and controls in terms of OOE, results show a large difference (REMS+experiencing 1.8 times higher costs than controls).

Results show that males experience large healthcare costs compared with females. This is in line with a previous study (conducted in Malawi) showing gender differences in health-seeking patterns.^27^ Among the lifestyle variables, drinking significantly affects healthcare and out-of-pocket expenditures. This is in line with evidence that current alcohol use is associated with less healthcare utilisation relative to abstainers generally (eg, the study conducted by Zarkin *et al*,^28^ considering a US population).

Our results are in line with studies estimating a large economic cost of MSK in high-income settings in terms of direct and indirect costs.^29 30^ Although estimates have limited comparability due to differences in population-level socioeconomic characteristics and healthcare systems, we reckon the actual burden of MSK to be more substantial in developing countries. Indeed, MSK disorders limit mobility and function, thus affecting particularly productivity and healthcare burden in those countries, such as Tanzania, with a large prevalence of manual workers. Furthermore, the absence of UHC leads to a strong reliance on OOE, putting financial pressure on households.

We acknowledge some limitations in our study. In the absence of a univocal correspondence between resource use and unit costs, we have used unit costs from NHIF, making assumptions on the type of facility (government/private/faith based), for example, assuming that all the hospital admissions took place in government facilities. This may have led to an underestimation of the actual costs. Self-reported costs may also have been affected by recall bias. The analysis of catastrophic expenditures presented some weaknesses, including the high percentage of missing responses in key variables (eg, food security, medical loans and assets) and a categorical (vs continuous) income variable. Also, as we did not include a question on food expenditure, we could not use the capacity to pay the definition of catastrophic expenditure. Furthermore, the matching between those with likely MSK and controls has been done on the basis of age and gender. While we control for relevant sociodemographic covariates in the regression analysis, we acknowledge that some relevant confounders might not have been included.

This study has revealed the significant economic burden associated with MSK conditions in Tanzania, filling an important research gap. Indeed, with previous research showing evidence of the burden posed by other NCDs^31^ (eg, cardiovascular, diabetes, cancer), the role played by MSK disorders in affecting costs to the healthcare system (ie, NIHF) as well as households’ financial burden had been neglected.

The results will be used to guide clinical health practices, intervention design, service provision and health promotion and awareness activities both at regional and national level.

Indeed, the large societal cost attributable to MSK claims the need for better integrating management of this condition into routine clinical care, strengthening medical capacity in order to provide specialist treatment, management and prevention services. Also, further research is advocated to explore effective and cost-effective preventative interventions, that could be implemented with success in the Tanzania setting and likely in the sub-Saharan setting.

Investing in the prevention, treatment and management of MSK disorders should be an urgent healthcare priority for Tanzania and other sub-Saharan African countries given the significant detrimental economic impacts associated with the clinical, disability and quality of life burden. In this regard, the dissemination of the results of this research to key stakeholders (community members, healthcare practitioners and policymakers) will increase awareness of the societal burden of MSK, fostering policy responses.

## supplementary material

10.1136/bmjopen-2024-087425 online supplemental file 1

## Data Availability

Data are available on reasonable request.
