# Supplementary material for: Economic burden of musculoskeletal disorders in Tanzania: results from a community-based survey
Source: BMJ Open. 2025 Jan 15;15(1):e087425. doi: 10.1136/bmjopen-2024-087425 (PMC11751939; doi:10.1136/bmjopen-2024-087425)
Supplement: online supplemental file 1 [file bmjopen-15-1-s001.docx]

**Appendix**

**CONTENT**

Supplementary Table 1 (Resource use and unit costs) ……………………………………………………....page 2

Supplementary Table 2 (Dependent and explanatory variables and corresponding survey questions) ……...page 3

Supplementary Table 3 (Missing values) ………………………………………………………………...… page 4

Supplementary Table 4 (Proportion of zero costs) …………………………………………………………. page 4

Supplementary Table 5 (Estimated yearly costs, complete-case analysis) …………………………………..page 5

Supplementary Appendix 6 (Income and Catastrophic expenditures)………………………………………page 6

**Supplementary Table 1: Resource use and unit costs**

| **Resource use** | **Measurement** | **Unit cost Int$ (source)** | |
| --- | --- | --- | --- |
| Outpatient visits to healthcare facilities |  |  |  |
| Government referral/specialised. Hospital | n. visits | 98.6 | Average cost per visit by level & ownership of the facility (government., private, NGOs), Tanzania mainland, NHIF unit cost 2017/2018^[[1]](#footnote-1)^, outpatient |
| Government regional hospital | n. visits | 36.6 |  |
| Government district hospital | n. visits | 19.3 |  |
| Government health centre | n. visits | 12.0 |  |
| Government dispensary | n. visits | 9.7 |  |
| Private specialised hospital | n. visits | 78.4 |  |
| Private health centre | n. visits | 16.9 |  |
| Private dispensary | n. visits | 14.1 |  |
| Pharmacy | n. visits | 35.8 |  |
| Total cost of visits to traditional healer or faith healer | NA (cost self-reported) | | |
| Inpatients admissions/stays |  |  |  |
| hospital admissions | n. admissions | 441.25 | Average cost per visit by level & ownership of the facility, Tanzania mainland, NHIF unit cost 2017/2018, inpatient GOVERNMENT TOTAL |
| Travel costs to the hospital where admission has been reported(round trip)-actual cost | NA (cost self-reported) | | |
| Travel costs to the hospital (round trip)-opportunity cost | n. hours evaluated using average wage | Average salary/day:  8.38 (Agriculture services)  9.64 (Trade, Industry and Commerce)  8.38 (Other) | Ministry of Labour and Employment^[[2]](#footnote-2)^ |
| Total costs of hospitalisation(s) in the hospital/medical facility | NA (cost self-reported) | | |
| Out-of-pocket expenses (including prescription medicines, tests, consultation and inpatient fees) | NA (cost self-reported) | | |
| **Loss of productivity** |  |  |  |
| Absenteeism (hours missed from work because of health problems) | no. hours | Average salary/day:  8.38 (Agriculture services)  9.64 (Trade, Industry and Commerce)  8.38 (Other) | Ministry of Labour and Employment |
| Presenteeism (impact on their work performance and “home-based” daily activities due to health problems ) | Percentage | Average salary/day:  8.38 (Agriculture services)  9.64 (Trade, Industry and Commerce)  8.38 (Other) | Ministry of Labour and Employment |

**Supplementary Table 2: Dependent and explanatory variables and corresponding survey questions**

| **Variable** | **Survey question** |
| --- | --- |
|  |  |
| **Explanatory variables** |  |
| Age | Age in years |
| Gender | Gender |
| Religion | What is your religion? (Christian/muslim/no religion/other) |
| Education | What is the highest level of school that you completed?primary/middle/higher |
| Family members experience of joint pain | Have others in your family experienced joint pain? |
| Smoking habits | Do you currently smoke or have you ever smoked? (Yes, current smoker/Yes, former smoker/No, never smoker) |
| Drinking habits | Do you currently drink alcohol or have you ever drunk alcohol? (Yes, Current drinker)/ Yes, Former drinker)/No, Never drinker) |
| Pregnancies | Have you ever been pregnant? |
| Diabetes | Has a doctor ever told you that you have diabetes? |
|  |  |
| **Dependent variables** |  |
| Absenteeism | In your/person opinion, during the past 7 days, how much did your/person health problems affect your ability to work while you were working? |
| Presenteeism | In your/person opinion, during the past 7 days, how much did your/person health problems affect your ability to work while you were working?  In your/person opinion, during the past seven days, how much did your/person health problems affect your ability to perform your normal “home-based” daily activities (e.g. walking, dressing, cleaning, collecting firewood, collecting water, cooking etc.), excluding the primary and secondary economic activities)? |
| Outpatients visits | During the past 3 months, what type of health facility did you /[person] visit and how was the treatment financed? |
| visits to faith/traditional healer | What were the total costs of your visit(s) to the traditional healer or faith dwellings for the past TWELVE months? |
| Hospital overnight stays | You mentioned earlier that you have been hospitalised/stayed over night in a hospital in the past TWELVE MONTHS.  How many admissions to the hospital did you have? |
| Hospital overnight stays (self-reported cost) | What were the total costs of your hospitalisation(s) or admission(s) in the hospital/medical facility? |
| self reported transport costs | Did you have to pay for the journey to the hospital(s) (e.g. health facility/center, district or regional hospital) in a round trip?  How much did the entire trip(s) cost? |
| opportunity cost of travel | How long did the journey take to go from your home to the hospital(s) (e.g. health facility/centre, district of regional hospital) in a round trip?  (days/hours/minutes) |
| Out-of-pocket expenditure | How much in total did the household spend on you in the past 4 weeks for all illnesses and injuries. Including for prescription medicines, tests, consultation and inpatient fees, if any? |

**Supplementary Table 3: Missing values**

| **Dependent variables** |  |  |  |  |
| --- | --- | --- | --- | --- |
|  | REMS+ | Controls | Total | %TOT missingness |
| Productivity | 79 | 119 | 198 | 33.33% |
| Healthcare costs (I) | 0 | 2 | 2 | 0.34% |
| Healthcare costs (II) | 0 | 2 | 2 | 0.34% |
| Healthcare costs (III) | 0 | 2 | 2 | 0.34% |
| Out of pocket expenditures | 57 | 83 | 140 | 23.57% |
| **Explanatory variables** |  |  |  |  |
|  | REMS+ | Controls | Total | %TOT missingness |
| Family members experience joint pain | 19 | 86 | 105 | 17.68% |
| Smoking | 1 | 63 | 64 | 10.77% |
| Drinking | 1 | 63 | 64 | 10.77% |
| Pregnancies | 1 | 44 | 45 | 7.58% |
| Diagnosis of diabetes | 1 | 63 | 64 | 10.77% |
| Note: age, sex, religion, education, marital status have 0 missings | | | | |

**Supplementary Table 4: Proportion of zero costs**

|  | MSK group | | Control group | | Total |  |
| --- | --- | --- | --- | --- | --- | --- |
| **Cost category** | **no. zero** | **% (over complete case)** | **no. zero** | **% (over complete case)** | **no. zero** | **% (over complete case)** |
| Productivity costs | 1 | 2% | 121 | 42% | 122 | 35% |
| Healthcare costs (I) | 38 | 63% | 245 | 86% | 283 | 82% |
| Healthcare costs (II) | 37 | 62% | 244 | 86% | 281 | 81% |
| Healthcare costs (III) | 38 | 63% | 245 | 86% | 283 | 82% |
| Out of pocket expenditures | 17 | 28% | 222 | 78% | 239 | 69% |

**Supplementary Table 5: Estimated yearly costs, complete- case analysis**

| **Predicted costs (Complete case dataset analysis)** | | |
| --- | --- | --- |
| **Average annual cost/individual, by MSK status** | | |
| Group | **Productivity costs (Int $)** | **95% Confidence intervals** |
| Control | 107 | (81, 134) |
| REMS+ | 447 | (334, 560) |
|  | **Healthcare costs (I)(Int $)** |  |
| Control | 53 | (32, 74) |
| REMS+ | 151 | (55, 248) |
|  | **Out-of-pocket expenses(Int $)** |  |
| Control | 167 | (-303, 637) |
| REMS+ | 577 | (-1541, 2694) |
|  |  |  |
|  | **Healthcare costs (II)(Int $)** |  |
| Control | 45 | (25, 65) |
| REMS+ | 182 | (66, 299) |
|  |  |  |
|  | **Healthcare costs (III)(Int $)** |  |
| Control | 508 | (211, 805) |
| REMS+ | 740 | (130, 1350) |

**Supplementary Appendix 6**

For catastrophic health expenditure (CHE), two definitions are often used: a)health spending exceeding 10% of total household resources (ability to pay), or b) health spending exceeding 40% of total non-food household expenditure (capacity to pay)[1]. Here, the capacity to pay equals income left after paying for food. In the community survey we did not collect food spend; therefore, we used the ability to pay definition.

We estimated out-of-pocket costs, reported as total spent in last month on healthcare, as a percentage of income. As the survey question gave income ranges as response options, the average and maximum point in each income range was converted to international dollars using the Purchasing Power parity. There was a high degree of missingness to the question on income (41%). A regression approach, in consideration of the missingness rate in the question on out of pocket expenditures and in the potential explanatory variables, was deemed inappropriate, considering the overall degree of missingness. A descriptive analysis on both complete case and multiple imputation was undertaken. Whilst absolute mean values differed using imputed or complete case dataset the relationship held that people with confirmed MSK disorders have a lower income on average and are more likely to incur higher out-of-pocket costs, than those who do not have MSK. Participants with MSK spend on average more than 10% of their total income on healthcare. The World Health Organisation refers to this level of expenditure as ‘catastrophic’ which means they suffer undue financial hardship. A significant different between those with MSK disorders and control was found, with approximately a tenth of the controls incurred catastrophic expenditure, and around a quarter of those with MSK disorders incurred catastrophic expenditure.

While results on income and catastrophic expenditures should be taken with caution, as the analysis is purely descriptive, they suggest a positive association between MSK and the probability to incur catastrophic expenditures. This suggest that higher healthcare expenditures, absence of universal health coverage and productivity impairments associated with MSK can be a predictor of impoverishment.

1. Wagstaff A. *Measuring financial protection in health*: World Bank Publications, 2008.

1. URT. National Health Insurance Fund (NHIF) Unit Cost for Mainland Tanzania and Zanzibar, 2017/18.; 2018. [↑](#footnote-ref-1)
2. Ministry of Labour and Employment; https://alrei.org/research/data-bases/minimum-wages/tanzania/archive-before-2019/minimum-wages-in-tanzania-with-effect-from-01-07-2013; own calculation. [↑](#footnote-ref-2)
