# Supplementary material for: Economic burden of musculoskeletal disorders in Tanzania: results from a community-based survey
Source: BMJ Open. 2025 Jan 15;15(1):e087425. doi: 10.1136/bmjopen-2024-087425 (PMC11751939; doi:10.1136/bmjopen-2024-087425)
Supplement: Supplementary file 2 [file bmjopen-2024-087425.reviewer_comments.pdf]

PEER REVIEW HISTORY

BMJ Open publishes all reviews undertaken for accepted manuscripts. Reviewers are asked to complete a checklist review form and are provided with free text boxes to elaborate on their assessment. These free text comments are reproduced below.

ARTICLE DETAILS

Title (Provisional)

The Economic burden of musculoskeletal disorders in Tanzania: results from a community-based survey

Authors

Deidda, Manuela; Grieve, Eleanor; Krauth, Stefanie; Hsieh, Ping-Hsuan; Yongolo, Nateiya; Siebert, Stefan; Halliday, J; Biswaro, Sanjura Mandela; Kilonzo, Kajiru; Walker, Richard; Kelly, Clive; Msoka, Elizabeth F.; Kiula, Kiula; Mmbaga, Blandina; McIntosh, Emma

---

VERSION 1 - REVIEW

**Reviewer**	**1**
**Name**	**Gorasso, Vanessa**
**Affiliation**	**Sciensano**
**Date**	**01-Jul-2024**
**COI**	**None**

Thank you for your paper. I found it very interesting and of utmost importance when it comes to healthcare provision and prevention. You will find below some remarks per section.

General:

The section summary should be called “abstract”.

There are some parts of the manuscript that are highlighted in grey, as well as some references.

Please add the currency used in your tables reporting costs.

Do I understand correctly that you use being REMS+ as a synonym for having MSK disorders? It is very confusing so I would recommend using only one definition. If being REMS+ is your definition of MSK disorder then you can use only the wording “having MSK disorders” or the other way around.

Methods:

• When you described the study design of your study it could be interesting to have a figure that would facilitate the understanding of cases and controls and the fact that is a matched study.

• In the supplementary table 1 could you please provide the exact reference for the unit costs? i.e. the website/report/publication where these numbers can be found.

• Were there any other confounders included in the economic model? For now you are only adjusting for the variables you used to match the samples. This will probably have a big impact on the causal assumption of your estimates.

• Is it possible to present confidence intervals derived from the imputed datasets?

Results:

• How do you interpret the fact that total costs are not significant but costs by category are significantly different?

• Please re-write the description of Table 5 as now it is not very clear what you are showing. Which are the two models presented? Express this also in the title of the table.

• Was the difference in "catastrophic expenditure" significantly different?

Discussion:

• Matching of the study is not very strong – there are many confounders that were not taken into account. Please add a reflection of this limitation in the discussion section.

• References 27,28: for which countries are they performed? In many countries, women seek more often healthcare and alcohol consumers have higher healthcare costs due to the diseases linked to alcohol.

• Can you please elaborate on your proposition of the use of your study to "guide clinical health practices, intervention design, service provision, and health promotion and awareness activities"?

Reviewer 2

Name Sørensen, Jan

Affiliation The Royal College of Surgeons in Ireland, Healthcare Outcomes Research Center

Date 07-Jul-2024

COI None

This paper explores the cost (economic burden) of musculoskeletal disorders (MSD) in a region of Tanzania based on a community-based cross-sectional survey. Respondents with MSD were identified using clinical screening tools, and controls were matched 1:3 by age and gender. Participants completed a cost questionnaire with different sections including

work productivity and use of healthcare resources. The analysis follows standard guidelines and practices for cost analyses including two-phased modelling of different cost components.

This manuscript builds on previous manuscripts including a published protocol (ref 12) and an unpublished prevalence study (ref 13). There may be some overlap between these papers, but this manuscript and ref 13 appear to be reporting on different aspects of the survey. The extent of overlap has not been explored due to the lack of access to ref 13.

In this manuscript, it is not entirely clear, how participants/households were recruited. This paper states that the sample of participants is representative, but unspecific in which aspects. The substantial level of non-respondents (594 out of 5701 individuals ~ inclusion rate of around 10%) has not been explored in the formal analysis nor commented upon in the discussion. This may have been included in the previous paper on prevalence, but should at least be mentioned in the current paper.

The paper states that no or little information about the economic burden of MSD in Tanzania is available. This is probably true, and therefore the paper offers new information and explores methods for obtaining information about burden of diseases in Tanzania.

The paper is a bit unclear about the unit of analysis. Presumably it is individuals with MSD and the aim is to identify costs attributable to MSK. The text is inconsistent by mentioning "household costs" several times, and also adjusts for household clustering effects. For the latter, it is unclear how many respondents might be living in the same household. This could be a relevant information to mention, especially because the authors have chosen to adjust for clustering effects in their analysis. In which case, it might also be relevant to mention the distribution of case and control participants in households – i.e. how many household members were deemed to have MSD.

The paper includes a consideration of "catastrophic expenditures". This is potentially relevant information. However, the authors are concerned about the validity of the data – more than 30% of respondents failed to provide information about (household?) income, and other relevant household variables were also missing. I am wondering, if the paper will improve in clarity, if the analysis of catastrophic expenditures were removed from the main text (currently only mentioned a few places) and then provide a longer discussion section (or supplementary material) which would report on the relevance, data quality, summary findings and interpretation. This would be easy to implement as the text related to catastrophic expenditures is relatively limited and described in small, easily identifiable paragraphs.

The cost data were adjusted to 2020-values. I am wondering why the costs were not updated to more recent price-levels. However, it is clearly stated in the text (as all the other relevant adjustments). The full adjustment procedure should be included explicitly in the relevant table headings to avoid misunderstanding.

The statistical analysis uses a two-phased approach. This is a good choice to handle the presence of many instances with no cost. This analytical approach is also called hurdle regression and is a standard procedure in Stata (churdle). The advantage of the churdle procedure is that the variation is considered concurrently and margins procedures are readily available. The authors have chosen to include all exogenous variables in both the logistic and GLM models. This is not necessarily a problem, but the statistical power could perhaps be strengthened, if the selection model (logistic regression) had less or different variables that needed for the GLM model. Certainly, the explanatory power of the logistic model seems poor.

Further, the authors have omitted to report statistical variation of 95% confidence intervals in the tables with cost differences (Table 3 and 5) and these would be readily available from the margins command following the churdle estimation.

The main results text could use the term "attributable cost" and include measures of statistical variation (95% CI).

The discussion section could include a section about the different nature of productivity loss and healthcare costs and potential concerns one might have by adding them together as a "total cost". They are different in nature and some economists recommend separate reporting only.

Tables:

Generally, the tables are not publication ready. They appear as Excel tables and do not follow journal style.

The use of variable names rather than full text is not good and should be changed throughout all tables. The footnote explanations are not reader friendly. An ideal to aim for would be that the tables should be independently understandable and interpretable. At the moment they are not.

Tab 1: I am wondering about the relevance and validity of the total cost block. I think it can be deleted as irrelevant.

Tab 2: I am wondering if "Total cost" includes or excludes out of pocket expenses.

Why is age included as continuous variable rather than categorical age groups?

Could provide some explanation as to the construction of the total cost – presumably it includes all cost components with missing data included as zero use or imputed by multiple imputation.

Table heading could indicate handling of missing data.

Tab 3: Would be good to have some measure of variation or 95% CI.

Also, include the attributable cost – i.e. difference between REMS and controls including 95%CI

Work on the table layout. Could perhaps be combined with Tab 5.

Tab 4: Work on table title and labels – what is "HC COST (II)" ? – comparable with "Total cost" in Tab 2?

Tab 5: As mentioned under Tab 3. Perhaps this table should be presented after the current Tab 2 and Tab 4 with the estimations.

Supp Tab 2. Requires some editing. Use the variable labels consistent in all tables.

Supp Tab 4: Probably provide separate numbers for REMS and controls

Supp Tab 5: as Tab 3 and 5 above.

VERSION 1 - AUTHOR RESPONSE

Reviewer: 1
Dr. Vanessa Gorasso, Sciensano Comments to the Author:

- Thank you for your paper. I found it very interesting and of utmost importance when it comes to healthcare provision and prevention. You will find below some remarks per section.

Answer: Thank you very much for your positive feedback.

General:

- The section summary should be called "abstract".

Answer: this has been changed in the revised manuscript.

- There are some parts of the manuscript that are highlighted in grey, as well as some references.

Answer: thanks for pointing this up. There was a formatting issue that has been solved in the revised version of the manuscript.

- Please add the currency used in your tables reporting costs.

Answer: thanks, we have added the currency in the column heading in Tables 3 and 5.

- Do I understand correctly that you use being REMS+ as a synonym for having MSK disorders? It is very confusing so I would recommend using only one definition. If being REMS+ is your definition of MSK disorder then you can use only the wording "having MSK disorders" or the other way around.

Answer: thanks for your comment, as explained on page 3 REMS+ implied confirmed MSK disorders. We have consistently used ‘MSK’ or ‘having MSK disorders’ in the revised manuscript.

Methods:

- When you described the study design of your study it could be interesting to have a figure that would facilitate the understanding of cases and controls and the fact that is a matched study.

Answer: thank you, we have added a sentence on page 3 to include the numbers of cases (MSK) and control individuals. The sentence reads as ‘Overall, the study included 153 REMS+ individuals (i.e. with likely MSK disorders) and 441controls.’

- In the supplementary table 1 could you please provide the exact reference for the unit costs? i.e. the website/report/publication where these numbers can be found.

Answer: thank you, we have added the references for the unit costs in the supplementary material. Please note that a list of NHIF unit costs for outpatients visits and hospital stays have been provided by the colleagues based in Tanzania and is not available online.

- Were there any other confounders included in the economic model? For now you are only adjusting for the variables you used to match the samples. This will probably have a big impact on the causal assumption of your estimates.

Answer: The matching between individuals with MSK and controls was done on the basis of age and gender. However, we have adjusted the cost regression for relevant socioeconomic covariates. As explained on page 5 *“Regressions were also adjusted by demographics (age, age squared, gender), socio-economic (religion, education, occupation, marital status) and clinical/lifestyle (family members who had experienced joint pain, smoking and drinking habits, pregnancies, diagnosis of diabetes) variables.”*

- Is it possible to present confidence intervals derived from the imputed datasets? **Answer:** we have now added confidence intervals in the tables presenting the cost estimates (Table 3 and Table 5).

Results:

- How do you interpret the fact that total costs are not significant but costs by category are significantly different?

Answer: As shown in table 2, only out of pocket expenses are not significant, whereas loss of productivity, healthcare costs and total costs are significantly different between groups. Please note that following the suggestion of the other referee the ‘Total cost’ category has been removed.

- Please re-write the description of Table 5 as now it is not very clear what you are showing. Which are the two models presented? Express this also in the title of the table.

Answer: table 5 shows the Average annual costs per participant, for MSK individuals and controls, i.e. this is the predicted cost estimate from the regression presented in table 4, adjusted by the covariates included in the regression. The title of Table 5 has been changed as "Average annual cost, per participant, by MSK/Control" , while the table title has been changed as "Predicted costs (Multiply imputed dataset analysis)".

- Was the difference in "catastrophic expenditure" significantly different?

Answer: yes, the difference was statistically significant; this has been clarified in the section on catastrophic expenditures (please note the methods and results on catastrophic expenditures have been moved to the supplementary material, following the suggestion of the other referee).

<u>Discussion:</u>

- Matching of the study is not very strong – there are many confounders that were not taken into account. Please add a reflection of this limitation in the discussion section.

Answer: thanks for your suggestion. We have added the following sentence in the discussion section *" Furthermore, the matching between those with likely MSK and controls has been done on the basis of age and gender. While we control for relevant socio-demographic covariates in the regression analysis, we acknowledge that some relevant confounders might not have included."*

- References 27,28: for which countries are they performed? In many countries, women seek more often healthcare and alcohol consumers have higher healthcare costs due to the diseases linked to alcohol.

Answer: Reference 27 considers a Malawi population, while reference 28 considers a US context.

- Can you please elaborate on your proposition of the use of your study to "guide clinical health practices, intervention design, service provision, and health promotion and awareness activities"?

Answer: thanks for your comment. We have included a revised and expanded section in the dicusssion section (page 8) *'The results will be used to guide clinical health practices, intervention design, service provision, and health promotion and awareness activities both at regional and national level. Indeed, the large societal cost attributable to MSK claims the need for better integrating management of this condition into routine clinical care, strengthening medical capacity in order to provide specialist treatment, management and prevention services. Also, further research is advocated to explore effective and cost-effective preventative interventions, that could be implemented with success in the Tanzania setting and likely in the Sub-Saharan setting. Investing in the prevention, treatment and management of MSK disorders*

should be an urgent health care priority for Tanzania and other sub-Saharan African countries given the significant detrimental economic impacts associated with the clinical, disability and quality of life burden. In this regards, the dissemination of the results of this research to key stakeholders (community members, healthcare practitioners and policymakers) will increase awareness on the societal burden of MSK, fostering policy responses.'

Reviewer: 2
Prof. Jan Sørensen, The Royal College of Surgeons in Ireland, Syddansk Universitet
Comments to the Author:

- This paper explores the cost (economic burden) of musculoskeletal disorders (MSD) in a region of Tanzania based on a community-based cross-sectional survey. Respondents with MSD were identified using clinical screening tools, and controls were matched 1:3 by age and gender. Participants completed a cost questionnaire with different sections including work productivity and use of healthcare resources. The analysis follows standard guidelines and practices for cost analyses including twophased modelling of different cost components.
  This manuscript builds on previous manuscripts including a published protocol (ref 12) and an unpublished prevalence study (ref 13). There may be some overlap between these papers, but this manuscript and ref 13 appear to be reporting on different aspects of the survey. The extent of overlap has not been explored due to the lack of access to ref 13.

Answer: thanks for your comment, the prevalence paper has been finalised and will be submitted soon. To date, it is still unpublished.

- In this manuscript, it is not entirely clear, how participants/households were recruited. This paper states that the sample of participants is representative, but unspecific in which aspects. The substantial level of non-respondents (594 out of 5701 individuals ~ inclusion rate of around 10%) has not been explored in the formal analysis nor commented upon in the discussion. This may have been included in the previous paper on prevalence, but should at least be mentioned in the current paper.

Answer: thanks for your comment, and we apologise if the recruitment strategy was not clear in the previous version of the manuscript. We have revised the section on methodscommunity survey to clarify the sampling strategy and the numbers of households and individuals included in the study. The revised section reads as follows: *"A community-based cross-sectional survey was undertaken between January 2021 and September 2021 in the Kilimanjaro region of Tanzania. We selected households using a two-stage random cluster sampling. In the first stage, 12 villages were selected with replacement and probability proportional to the size. In the second stage, 70 households per village were randomly selected. Overall, 1105 households were approached; 45 households declined participation and 40 households were not reached. Within 1020 participating households, 2982 individuals were eligible for participation. After excluding those who could not be reached and those who declined participation, a sample of 2632 individuals went through a tiered process. Clinical screening tools, including the Gait*

Arms Legs Spine (GALS)[11] and Regional Examination of the Musculoskeletal system (REMS)[10], were used to screen people with likely MSK diseases and possible arthritis. In the first tier, participants underwent GALS examination. Participants who were positive on GALS screening (222) underwent subsequent REMS (tier 2). 154 participants were positive on REMS assessment (REMS+, implying confirmed MSK disorders). Controls were selected from the GALS- group, and matched on age and gender on a 1: 3 ratio. REMS+ and controls were administered a detailed health economics questionnaire.
Overall, the study included 153 individuals with likely MSK disorders (REMS+) and 441controls."

The paper states that no or little information about the economic burden of MSD in Tanzania is available. This is probably true, and therefore the paper offers new information and explores methods for obtaining information about burden of diseases in Tanzania.
Answer: indeed, at the time of the analysis, there was lack of data on the prevalence and impact of MSK in terms of economic burden and quality of life Tanzania.

- The paper is a bit unclear about the unit of analysis. Presumably it is individuals with MSD and the aim is to identify costs attributable to MSK. The text is inconsistent by mentioning "household costs" several times, and also adjusts for household clustering effects. For the latter, it is unclear how many respondents might be living in the same household. This could be a relevant information to mention, especially because the authors have chosen to adjust for clustering effects in their analysis. In which case, it might also be relevant to mention the distribution of case and control participants in households – i.e. how many household members were deemed to have MSD.
  Answer: the unit of analysis is the individual. All the questions (healthcare utilization, productivity) were asked to each eligible individual living in the household. However, questions on income and wealth were asked to the financial respondent. This has been clarified on page 4, where the following paragraph has been included *"All eligible individuals within selected households in the MSK and control group respondent to questions on labour, productivity, health and healthcare utilization. Questions on income and wealth were answered by the person identified as the final respondent (i.e. the person who has the final decision regarding the household finances)."* We apologise if this was not clear in the previous version of the manuscript.

On page 5 we have added the following sentence: *"In 99 households two individuals were selected from the same household; in 5 households three individuals were selected from the same households. In the households where more than one individual was selected, all households members were found as having MSK disorders in 60 instances; in 10 households, all households members did not have MSK."*

- The paper includes a consideration of “catastrophic expenditures”. This is potentially relevant information. However, the authors are concerned about the validity of the data – more than 30% of respondents failed to provide information about (household?) income, and other relevant household variables were also missing. I am wondering, if the paper will improve in clarity, if the analysis of catastrophic expenditures were removed from the main text (currently only mentioned a few places) and then provide a longer discussion section (or supplementary material) which would report on the relevance, data quality, summary findings and interpretation. This would be easy to implement as the text related to catastrophic expenditures is relatively limited and described in small, easily identifiable paragraphs.

Answer: thanks for your suggestion. We have moved the text on catastrophic expenditures in supplementary appendix 6.

- The cost data were adjusted to 2020-values. I am wondering why the costs were not updated to more recent price-levels. However, it is clearly stated in the text (as all the other relevant adjustments). The full adjustment procedure should be included explicitly in the relevant table headings to avoid misunderstanding.

Answer: thank you for your comment. We added the following to Tables 1, 3 and 5 “All costs were inflated to 2020 values, scaled to 1 year and converted to international dollars using the purchasing power parity exchange rate”. The table heading also mentions PPP and inflation adjustment (e.g. in Table 3 Productivity costs (Int $, 2020))

- The statistical analysis uses a two-phased approach. This is a good choice to handle the presence of many instances with no cost. This analytical approach is also called hurdle regression and is a standard procedure in Stata (churdle). The advantage of the churdle procedure is that the variation is considered concurrently and margins procedures are readily available. The authors have chosen to include all exogenous variables in both the logistic and GLM models. This is not necessarily a problem, but the statistical power could perhaps be strengthened, if the selection model (logistic regression) had less or different variables that needed for the GLM model. Certainly, the explanatory power of the logistic model seems poor.
  Further, the authors have omitted to report statistical variation of 95% confidence intervals in the tables with cost differences (Table 3 and 5) and these would be readily available from the margins command following the churdle estimation.
  The main results text could use the term “attributable cost” and include measures of statistical variation (95% CI).

Answer: thank you very much for your comment. Indeed, both hurdle regression and twopart models can account for a large mass of zeros in the dependent variable, and are suited to model cost variables. Specifically, in the two-part model, a logistic regression estimates the probability of observing a non-zero outcome, and a GLM is fitted for the positive outcome,

conditional on a positive outcome. We have chosen to run a two-part model using the STATA command, *twopm* (Belotti et al, 2015[1]) as this allows flexibility in the choice of the appropriate combination of link and family. This is of utmost importance for healthcare cost data, that are right skewed.

Also, *twopm* can calculate marginal effects (and their standard errors) from the combined first- and second-part models. In the revised Table 3 and Table 5 we have included the 95% confidence intervals.

Finally, *twopm* allows the inclusion of different explanatory variables in the first and second part. We tested different specifications, and result did not change substantially. Also, we agreed with the project team that it was reasonable to assume that the same determinants of healthcare utilization are also affecting the magnitude of costs.

- The discussion section could include a section about the different nature of productivity loss and healthcare costs and potential concerns one might have by adding them together as a "total cost". They are different in nature and some economists recommend separate reporting only.

Answer: Thanks for your comment. Following your comment below we have deleted the total block from Table 1 and the regressions.

- Tables:

  Generally, the tables are not publication ready. They appear as Excel tables and do not follow journal style.

  The use of variable names rather than full text is not good and should be changed throughout all tables. The footnote explanations are not reader friendly. An ideal to aim for would be that the tables should be independently understandable and interpretable. At the moment they are not.

  Tab 1: I am wondering about the relevance and validity of the total cost block. I think it can be deleted as irrelevant.

  Tab 2: I am wondering if "Total cost" includes or excludes out of pocket expenses.

  Why is age included as continuous variable rather than categorical age groups?

  Could provide some explanation as to the construction of the total cost – presumably it includes all cost components with missing data included as zero use or imputed by multiple imputation.

  Table heading could indicate handling of missing data.

  Tab 3: Would be good to have some measure of variation or 95% CI.

  Also, include the attributable cost – i.e. difference between REMS and controls including 95%CI

  Work on the table layout. Could perhaps be combined with Tab 5.

---

[1] Belotti, Federico, et al. "twopm: Two-part models." The Stata Journal 15.1 (2015): 3-20.

Tab 4: Work on table title and labels – what is “HC COST (II)” ? – comparable with “Total cost” in Tab 2?

Tab 5: As mentioned under Tab 3. Perhaps this table should be presented after the current Tab 2 and Tab 4 with the estimations.

Supp Tab 2. Requires some editing. Use the variable labels consistent in all tables. Supp Tab 4: Probably provide separate numbers for REMS and controls Supp Tab 5: as Tab 3 and 5 above.

Answer: thank you very much for your suggestions, in the revised manuscript we have incorporated your feedback:

o We have improved the tables format, aligning them to the journal’s formatting guidance

o We have changed the variables’ name, renaming them when necessary eg. variable rems_ctrl (dummy=1 if the individual has MSK disorders) has been renamed as ‘MSK’; HC_cost1 has been renamed as Healthcare costs (I), and a definition has been provided in the footnote explanations. We have used consistent variables name throughout the paper.

o Following your suggestion, the variable ‘Total costs’ has been removed. Total costs did not include out of pocket expenses. As explained on page 5 (previous submission) total costs were calculated as the sum of. productivity and healthcare costs (sum of total costs for outpatient visits, visits to faith/traditional healer, hospital overnight stays (calculated multiplying the number of admissions by the NHIF unit cost), self-reported actual transport cost).

o We have used a continuous age variable, plus a quadratic term, to model the non-linear relation between cost variables and age.

o We have included 95% confidence intervals in Tables 3, 5 and supplementary Table 5.

We have provided separate numbers by category (MSK and controls).

VERSION 2 - REVIEW

Reviewer 2

Name Sørensen, Jan

Affiliation The Royal College of Surgeons in Ireland, Healthcare Outcomes Research Center

Date 12-Nov-2024

COI

I appreciate the detailed changes made as suggested in the previous reviews.

The manuscript is now a very tight, clear and informative report.

The data capturing process is clearly described and the statistical analysis is advanced and appropriate for the data including two-phased modelling and appropriate handing of missing information.

The use of supplementary material is good and the tables are clear and easy to understand.

I am still wondering about the catastrophic expenditures. They have been analysed in appendix 6 and that is appropriate. They are also referred to in the abstract, but not in the method, result or discussion sections (as far as I can see).

A few small suggestions:

Abstract:

Authors report p-value for cost differences rather than bootstrap confidence intervals, which might convey more information and follow standard recommendations – Ramsey et al, VIH, 2015 p 166, 2. 2. Paragraph under trial cost. These have been provided in Table 3, where the statistical difference is less apparent.

L36: currency is missing before 343. Why was p-value not provided here?

Introduction:

Full stop and commas need editing – line 15-17 – drop full stop after diagnosis – probably replace with comma. Include full stop after safety net.

Community survey:

L 49: include sampling "strategy" or edit sentence otherwise

P5 L7: repetition of sentence above

Healthcare and productivity costs:

P7 l10: Table 1 shows

L 33: check/edit statement: "Being a current drinker, as well as having a diagnosis of diabetes, is negatively associated with loss of productivity costs". Suggest that drinkers has higher productivity costs – perhaps avoid double negatives

Table 4. Is it correct that the estimates for both parts have the same value? Check if there is an error

---

VERSION 2 - AUTHOR RESPONSE

Reviewer: 2
Prof. Jan Sørensen, The Royal College of Surgeons in Ireland, Syddansk Universitet

- I appreciate the detailed changes made as suggested in the previous reviews. The manuscript is now a very tight, clear and informative report. The data capturing process is clearly described and the statistical analysis is advanced and appropriate for the data including two-phased modelling and appropriate handing of missing information. The use of supplementary material is good and the tables are clear and easy to understand.
  Answer: thanks, your comments were indeed very useful to improve the clarity of the paper.

- I am still wondering about the catastrophic expenditures. They have been analysed in appendix 6 and that is appropriate. They are also referred to in the abstract, but not in the method, result or discussion sections (as far as I can see).
- **Answer:** thanks for your comment, we have now referred to catastrophic expenditures in the methods and in the results, while presenting full details in the supplementary appendix. On page 6 we have added the following sentence: *"We analysed catastrophic expenditures, using the 'ability to pay' definition (i.e. health spending exceeding 10% of total household resources [26]). Full details on the methods used to estimate catastrophic expenditures have been presented in the supplementary material (Supplementary Appendix 6)."* We have also added a sentence on page 7*: "Results on catastrophic expenditures (Supplementary appendix 6) show that around a quarter of those with MSK disorders incurred catastrophic expenditure, vs approximately a tenth of the controls."*

  A few small suggestions:

- Abstract:
  Authors report p-value for cost differences rather than bootstrap confidence intervals, which might convey more information and follow standard recommendations – Ramsey et al, VIH, 2015 p 166, 2. 2. Paragraph under trial cost. These have been provided in Table 3, where the statistical difference is less apparent.
  L36: currency is missing before 343. Why was p-value not provided here?
  Answer: thanks for your suggestion. Please note that we have updated the results in Table 3 and Table 5 as we found a coding issue that was affecting the way margins were calculated, inflating costs in both groups. This did not affect regression results (Table 2 and Table 4), but only the predicted costs for MSK group and control (Table 3 and Table 5). The overall results and key message of the paper (i.e. statistical significance of productivity and healthcare cost difference) do not change.
  In the revised manuscript we have included the difference in costs alongside the predicted cost by group (MSK vs. control), and the frequentist confidence interval. While the ISPOR Task Force report from Ramsey et al (2015) suggests using bootstrapped confidence intervals to compare means from trial cost, they also raise concerns on the superiority of bootstrapping(page 166) '*In most cases, the nonparametric bootstrap is an appropriate method to compare means and calculate confidence intervals although parametric methods provide a unique solution and have been shown to provide coverage equal or superior to that provided by bootstrap methods'*. Also, Ramsey et al (2015) is focused on economic evaluations alongside clinical trials, while our analysis is a costing analysis conducted alongside survey data.
  We agree with your suggestion of including confidence intervals, rather than p-values, for cost differences. We have now revised the sentence in the abstract as follows *"Annual average productivity and healthcare costs were, respectively, 3·5 and 3 times higher for those with likely MSK disorders than controls. Productivity costs of individuals with MSK disorders were Int$ 487, vs. Int$ 132 in the control group (Difference: Int$ 355, 95% CI: Int$ 222 to Int$ 488). Healthcare costs in those with MSK were Int$ 269 vs. Int $ 88 in the control group(Difference: Int$ 181, 95% CI: Int$ 34 to Int$ 327). The difference in terms of out-of-pocket expenses was economically substantial in magnitude, although not statistically significant."*

- Introduction:
  Full stop and commas need editing – line 15-17 – drop full stop after diagnosis – probably replace with comma. Include full stop after safety net.
  Answer: thanks, we have proofread the revised manuscript and checked the punctuation.

- Community survey:
  L 49: include sampling "strategy" or edit sentence otherwise
  Answer: thanks, the revised sentence now reads as "We selected households using a two-stage random cluster sampling strategy[12]"

- P5 L7: repetition of sentence above
  Answer: thanks for your comment. This has been changed in the revised version of the manuscript. The revised sentence reads *as 'The first part of the model consisted of a logistic regression model to estimate the probability of having any type of expenditure. The second part of the model used a GLM with a gamma distribution and a log link to estimate the total costs conditional on individuals with positive expenditures. Using a GLM allows modelling of potential skewed distribution of costs.'*

- Healthcare and productivity costs:
  P7 l10: Table 1 shows
  Answer: thanks, this has been changed in the revised manuscript.

- L 33: check/edit statement: "Being a current drinker, as well as having a diagnosis of diabetes, is negatively associated with loss of productivity costs". Suggest that drinkers has higher productivity costs – perhaps avoid double negatives
  Answer: thanks, this has been changed. The revised sentence reads as *"Being a current drinker, as well as having a diagnosis of diabetes, is associated with higher productivity costs".*

- Table 4. Is it correct that the estimates for both parts have the same value? Check if there is an error
  Answer: yes, this is correct the estimated coefficients (considering 6 decimals) are 0.706739 (first part) and 0.707380 (second part).
